# Expression profiling of spinal cord dorsal horn in a rat model of complex regional pain syndrome type-I uncovers potential mechanisms mediating pain and neuroinflammation responses

**DOI:** 10.1186/s12974-020-01834-0

**Published:** 2020-05-23

**Authors:** Ruixiang Chen, Chengyu Yin, Qimiao Hu, Boyu Liu, Yan Tai, Xiaoli Zheng, Yuanyuan Li, Jianqiao Fang, Boyi Liu

**Affiliations:** 1 Department of Neurobiology and Acupuncture Research, The Third Clinical Medical College, Zhejiang Chinese Medical University, Key Laboratory of Acupuncture and Neurology of Zhejiang Province, 548 Binwen Road, Hangzhou, 310053 China; 2 Academy of Chinese Medical Sciences, Zhejiang Chinese Medical University, Hangzhou, 310053 China

**Keywords:** RNA-Seq, Pain, CRPS-I, Spinal cord dorsal horn, Inflammation, Inflammasome, Cytokine

## Abstract

**Background:**

Complex regional pain syndrome type-I (CRPS-I) is a progressive and devastating pain condition. The mechanisms of CRPS-I still remain poorly understood. We aim to explore expression profiles of genes relevant to pain and neuroinflammation mechanisms involved in CRPS-I.

**Methods:**

The rat chronic post-ischemic pain (CPIP) model that mimics human CRPS-I was established. RNA-sequencing (RNA-Seq), qPCR, Western blot, immunostaining, and pharmacological studies were used for profiling gene changes in ipsilateral spinal cord dorsal horn (SCDH) of CPIP model rat and further validation.

**Results:**

CPIP rats developed persistent mechanical allodynia in bilateral hind paws, accompanied with obvious glial activation in SCDH. RNA-Seq identified a total of 435 differentially expressed genes (DEGs) in ipsilateral SCDH of CPIP rats. qPCR confirmed the expression of several representative genes. Functional analysis of DEGs identified that the most significantly enriched biological processes of upregulated genes include inflammatory and innate immune response. We further identified NLRP3 inflammasome expression to be significantly upregulated in SCDH of CPIP rats. Pharmacological blocking NLRP3 inflammasome reduced IL-1β overproduction, glial activation in SCDH as well as mechanical allodynia of CPIP rats.

**Conclusion:**

Our study revealed that immune and inflammatory responses are predominant biological events in SCDH of CPIP rats. We further identified NLRP3 inflammasome in SCDH as a key contributor to the pain and inflammation responses in CPIP rats. Thus, our study provided putative novel targets that may help to develop effective therapeutics against CRPS-I.

## Introduction

Complex regional pain syndrome type-I (CRPS-I) is a progressive and devastating neuropathic pain condition that usually affects the limb and is not accompanied with a clinically verifiable nerve injury [1]. CRPS-I usually develops after an initial injury, which includes ischemia, soft tissue trauma, surgery, or fractures to the extremity [2, 3]. CRPS-I can develop into chronic pain state that severely affects the patient’s life quality [4]. There are currently no specific drugs approved for the treatment of CRPS. Conventional treatments for CRPS-I include physiotherapy, sympathetic blockade, corticosteroids, and non-steroidal anti-inflammatory drugs (NSAIDs) [5]. However, none of the above treatment options produce satisfactory relieving effects on CRPS-I, which makes it one of the most clinically challenging neuropathic pain conditions [6].

Currently, the mechanisms of CRPS-I are still not fully understood. Ischemia/reperfusion injury is among one of the major causes leading to CRPS-I [7, 8]. In order to get more understandings of the mechanisms of CRPS-I, Coderre et al. established a rat chronic post-ischemic pain (CPIP) model by applying prolonged hind paw ischemia and reperfusion to mimic CRPS-I [9]. The CPIP model displayed many CRPS-I-like symptoms, including early hind paw edema, hyperemia, and skin warmth, accompanied with long-lasting neuropathic pain conditions, including bilateral mechanical and thermal hypersensitivities [9]. With the aid of this animal model, it is proposed that central pain sensitization, reactive oxygen species increase, TRPA1 activation, etc. may contribute to CRPS-I pathology [10–12]. We recently contributed to these efforts by performing transcriptome profiling of the dorsal root ganglia (DRG) of CPIP model rat and identified potential peripheral pain mechanisms involved in CRPS-I [13]. Our recent work further identified the pain-sensing ion channel TRPV1 in the DRG neurons as a key target involved in mediating the pain symptoms of CPIP model rats [14].

The spinal cord dorsal horn (SCDH) receives pain signal inputs from the peripheral sensory neurons and plays a critical role in integrating pain signals and central pain sensitization. Non-neuronal cells, such as astrocytes and microglia, are activated in SCDH of CPIP model rats and produce pro-inflammatory mediators, such as some cytokines and chemokines that can modulate pain process [11, 15, 16]. These substances act on spinal nociceptive neurons to produce neuroinflammation and sensitize pain-related receptors or ion channels to initiate central pain sensitization [17]. In order to further explore the central mechanisms underlying CRPS-I, we proceeded to carry out genome-wide expression profiling of the ipsilateral SCDH of CPIP model rats and sham control rats using RNA-Seq. We identified a number of differentially expressed genes (DEGs). We further examined the molecular/cellular functions and the signaling pathways that these DEGs were involved in. We compared our findings with previously published datasets of neuropathic pain models and identified a core set of genes and pathways that extensively participated in CPIP and other neuropathic pain conditions. By analyzing the RNA-Seq dataset, we further identified NLRP3 inflammasome in SCDH is a key player in mediating the pain and inflammation responses in CPIP model rats. Our work uncovered the gene expression patterns in the SCDH of a rat model of CRPS-I, which is essential for understanding the neuroinflammation and pain mechanisms of CRPS-I. Our work may provide further insights into identifying novel and effective therapeutic targets against CRPS-I.

## Materials and methods

### Animals

Male Sprague-Dawley rats (8–10 weeks, 300–320 g) were purchased from Shanghai Laboratory Animal Center, Chinese Academy of Sciences and housed in the Laboratory Animal Center of Zhejiang Chinese Medical University accredited by the Association for Assessment and Accreditation of Laboratory Animal Care (AAALAC) under standard environmental conditions (12-h light-dark cycles and 24 ± 2 °C). Food and water were provided ad libitum. Rats were randomly allocated and 4 rats were housed per cage. The rats were given a minimum of 1 week to adapt to the new environment before the experiment.

### CPIP rat model establishment

CPIP was established via prolonged hind paw ischemia and reperfusion as described previously [9, 18]. Anesthesia was induced in all rats with an intraperitoneal injection of 50 mg/kg of sodium phenobarbital and was maintained with an infusion of sodium phenobarbital at 20 mg/kg/h. An O-ring with 7/32 internal diameter was tightly passed around the right hind limb just proximal to the ankle joint. The O-ring was then cut off 3 h later for reperfusion. Sham rats received the same anesthetic procedure but the ankle was surrounded with a cut O-ring which did not block blood flow.

### Determination of mechanical allodynia

Rats were habituated to the test environment daily for a consecutive 3 days before baseline test. Rats were individually placed in transparent Plexiglas chambers on an elevated mesh floor and were habituated for 30 min before the test. The mechanical hyperalgesia was determined using a series of von Frey filaments (UGO Basile, Italy) applied perpendicularly to the midplantar surface of the hind paws, with sufficient force to bend the filament slightly for 3–5 s according to methods we previously used [19, 20]. An abrupt withdrawal of the paw and licking and vigorously shaking in response to stimulation were considered pain-like responses. The threshold was determined using the up-down testing paradigm, and the 50% paw withdrawal threshold (PWT) was calculated by the nonparametric Dixon test [21]. Behavior tests were all conducted by an experimenter blinded to experimental conditions.

### Determination of the hind paw swelling

Swelling was observed as an increase in hind paw diameter, as measured by a digital caliper, and was calculated as the difference between the basal value and the test value as in our previous study [14]. Changes in paw diameter were shown as % increase in paw diameter and calculated as follows: % increase in paw diameter = (*D*_after_ − *D*_before_)/*D*_before_. Each rat was measured 3 times, and the mean value was calculated.

### Tissue collect and RNA extraction

The L4-6 spinal cord dorsal horn segments ipsilateral to the side of ischemia/reperfusion injury were harvested and used for generating libraries for RNA-seq. At day 7, rats were deeply anesthetized with sodium pentobarbital (40 mg/kg) and were perfused transcardially with 200 mL 0.9% NaCl (4 °C). After the perfusion, the L4-6 spinal segments were extracted by lumbar laminectomy, separated into left (contralateral) and right (ipsilateral) cord, and the ipsilateral dorsal horn was immediately preserved in the RNA later solution (Invitrogen, Carlsbad, USA). Total RNA was extracted using Trizol reagent (Invitrogen, Carlsbad, USA) according to the manufacturer's instructions with DNase I to degrade contaminating DNA. The purity and concentration of the samples was assessed by the Nanodrop Spectrophotometer (NanoDrop Products, CA, USA), and the RNA integrity was assessed by the Agilent 2100 Bioanalyzer (Agilent Technologies, Palo Alto, CA).

### Immunofluorescence staining

Rats were deeply anesthetized with sodium pentobarbital (50 mg/kg) and were perfused transcardially with 200 mL 0.9% saline (4 °C) followed by 200 ml of 4% formaldehyde. The ipsilateral L4-6 spinal cord was harvested (contralateral side of the spinal cord was labeled by piercing a needle into the anterior horn) and post-fixed in the same fixative for 4 h (4 °C) before transfer to 15% and 30% sucrose for 72 h for dehydration. Several days later, the spinal cord was serially cut into 25-μm-thick transverse sections on a frozen microtome (Thermo NX50, USA) and mounted on gelatin-coated glass slides as 6 sets of every 5th serial sections. All the slides were blocked with 5% normal donkey serum in TBST (with 0.1% Tween-20) for 1 h at 37 °C and then incubated overnight with corresponding primary antibodies. The primary antibodies used were mouse anti-GFAP (#c9205, Sigma) and mouse anti-OX42 (#ab1211, Abcam). The following day, the sections were rinsed with TBST (6 × 10 min) and incubated for 1 h with a mixture of corresponding secondary antibodies. Fluorescence images were captured by Nikon A1R laser scanning confocal microscope (Nikon, Japan). For quantitative fluorescence intensity analysis, uniform microscope settings were maintained throughout all image capture sessions. All stained sections were examined and analyzed in a blinded manner. Five images were randomly selected per rat tissue and averaged and then compared according to methods described in our previous studies [22, 23].

### RNA-Seq library establishment and RNA-Seq

Total mRNAs from 3 rats of each group were isolated and used to construct sequencing libraries. mRNA molecules were purified from total RNA using oligo (dT)-attached magnetic beads. mRNAs were fragmented into small pieces using fragmentation reagent. First-strand cDNA was generated using random hexamer-primed reverse transcription and then was followed by a second-strand cDNA synthesis. The synthesized cDNA was subjected to end-repair and then was 3′ adenylated. Adaptors were ligated to the ends of these 3′ adenylated cDNA fragments. This process is to amplify the cDNA fragments with adaptors from the previous step. PCR products are purified with the SPRI beads and dissolved in EB solution. The double-stranded PCR products were heat denatured and circularized by the splint oligosequence. The single-strand circle DNA (ssCir DNA) was formatted as the final library. Library was validating on the Agilent Technologies 2100 bioanalyzer. The library was amplified with phi29 to make DNA nanoball (DNB) which have more than 300 copies of one molecular. The DNBs were load into the patterned nanoarray and single end 50 bases reads were generated in the way of sequencing by synthesis. Finally, the fragments were enriched by PCR amplification to construct a library ready for sequencing using BGISEQ-500 by BGI (Shenzhen, China).

### Bioinformatics analysis

Primary sequencing data produced by RNA-Seq (raw reads) were subjected to quality control (QC). The information of total reads and mapping ratio reads were shown in Table 1. Raw data were filtered into clean reads by internal software SOAPnuke (version 1.5.2), as follows: Remove reads in which unknown bases (N) are more than 10%; Remove reads with adaptors; Remove low-quality reads (we define the low-quality read as the percentage of base which quality is lesser than 15 and greater than 50% in a read). QC of alignment was performed to determine if re-sequencing was needed. If the alignment result passed QC, downstream analysis including gene expression, differentially expressed genes, cluster analysis, Gene Ontology (GO) enrichment analysis, Kyoto Encyclopedia of Genes and Genomes (KEGG) pathway enrichment analysis, etc. was proceeded with.

**Table 1:** The information of total reads and mapping ratio for Sham and CPIP groups in RNA-Seq

Sample	Total raw reads (Mb)	Total clean reads (Mb)	Total clean bases (Gb)	Clean reads Q20 (%)	Clean reads Q30 (%)
SCDH model 1	21.94	21.92	1.1	98.56	92.23
SCDH model 2	21.94	21.91	1.1	98.58	92.27
SCDH model 3	21.94	21.9	1.1	98.72	92.73
SCDH sham 1	21.94	21.89	1.09	98.16	90.86
SCDH sham 2	21.94	21.89	1.09	98.06	90.5
SCDH sham 3	21.94	21.92	1.1	98.55	92.16

### Cluster analysis and screening of differentially expressed genes

Distances of expressed genes were calculated using the Euclidean method [24]. The sum of the squared deviations algorithm was used to calculate distance. The cluster analysis and heat map visualization of gene expression patterns were performed using the “pheatmap” package in the R software of Bioconductor. Differentially expressed mRNAs with statistical significance were identified through Scatter Plot filtering as we reported before [25]. The threshold required for the results to be considered significant was as follows: *q* value ≤ 0.01 and absolute value of |log_2_ (Fold Change)| ≥ 1.0 as in our previous study [13].

### Functional enrichment analysis of DEGs

Functional enrichment analysis was performed by functional annotation package “clusterProfiler” in R studio software (RStudio, Boston, MA). GO and KEGG enrichment analysis was also conducted. For each enriched function term, the *p* value of enriched functions and the *p* value by multiple testing corrections were calculated out by “clusterProfiler” package in R studio software. The GO functional and KEGG pathway enrichment analysis were performed for DEGs using the Data-base for Annotation, Visualization and Integrated Discovery (DAVID) online tools (http://www.geneontology.org and http://www.genome.jp/kegg).

### GSEA analysis

Gene set enrichment analysis (GSEA) was performed according to methods previously described [26]. The gene sets for astrocyte activation (systematic name, M23832), microglia activation (systematic name, M25223), and oligodendrocyte differentiation (systematic name, M15768) were downloaded from the Molecular Signatures Database v7.1 (https://www.gsea-msigdb.org/gsea/msigdb/index.jsp). A FDR ≤ 0.25 was adopted as the criterion for judging significance [26].

### Real-time quantitative PCR (qPCR) analysis

Total RNA was extracted from the dorsal horn tissue using Trizol reagent (Invitrogen, Carlsbad, USA) according to the manufacturer’s protocol. Primer sequences are listed in Table 2. qPCR was performed using the Fast Start Universal SYBR Green Master kit (TaKaRa Bio Inc, China) with 25 μl reaction system according to the manufacturer’s protocol by CFX96 Real-Time System (Bio-Rad, USA). Each reaction was performed in triplicates and normalized to *Gapdh* gene expression. The CT value of each well was determined using the CFX96 Real-Time System software and the average of the triplicates was calculated. The relative quantification was determined by ΔΔCT method [27, 28].

**Table 2:** The primers used in qPCR

Primers	Forward	Reverse	Amplicon size (bp)
Cxcl13	CGACTTTGAAAGGTTGCTTGTA	ACACTGGATGAATAGGAAACGT	219
Nlrp3	GAGCTGGACCTCAGTGACAATGC	ACCAATGCGAGATCCTGACAACAC	146
Reg3b	GCTCTCCTGCCTGATGCTCTTATC	AGGTGTCCTTCAGGTCTCTTCTGG	186
Itgam	GCATCAGTAGCCAGCATCAGTACC	CCGTCCATTGTGAGATCCTTGCC	119
C3	TCTGCCTATGCTGCCTTCAACAAC	TGAATCACTGGTCCGTCCTCCTG	188
Il-1β	CAACTGTTCCTGAACTCAACTG	GAAGGAAAAGAAGGTGCTCATG	281
LOC100911956	AGGCAGCAACAGTTATCGTGACTC	CCTCATCGCCACCGTTGTTCC	197
Fam111a	ACACCGAATCACAGCGACTTAACC	ACCAGCTCCTTGCTTGCTCAATG	143
Mfap4	CACCTCCTGACACTGAAGCAGAAG	CTCCGCACTGACCGCATTGG	120
Gapdh	GACATGCCGCCTGGAGAAAC	AGCCCAGGATGCCCTTTAGT	92

### Source of microarray data

Two independent datasets of neuropathic pain models were selected for the study: spared nerve injury (SNI) and chronic constriction injury (CCI) neuropathic microarray. The SNI datasets (GSE18803) were downloaded from Gene Expression Omnibus (GEO) dataset, at the website of https://www.ncbi.nlm.nih.gov/geo/. The CCI datasets comes from a recently published article on CCI sequencing [29]. Differentially expressed genes from these microarray datasets were screened based on criteria as *q* value ≤ 0.01 and absolute value of |log_2_ (Fold Change)| ≥ 1.0.

### Protein-protein interaction (PPI) network analysis

The Search Tool for the Retrieval of Interacting Genes (STRING) is used to provide information regarding predicted and experimental interactions of proteins and the prediction method of this database is from neighborhood, gene fusion, co-occurrence, co-expression experiments, databases, and text mining. By setting the combination score > 0.4 as the reliability threshold value, the web-based STRING database (http://string-db.org/) was used to produce PPI predictions after uploading the union gene list to the search bar [30]. Based on the interplayed relationships, a PPI network was established and then visualized using the Cytoscape software [31]. The connectivity degree of each protein, namely the number of proteins it connected, was calculated to evaluate its importance in this network.

### Drug application

The Specific NLRP3 inflammasome inhibitor MCC950 (APExBIO Technology, TX, USA) was prepared in stock in DMSO and diluted in PBS (1:1000) prior to use. It was applied via intrathecal catheter (30 μg/rat/day, 12.5 μl injection volume). Vehicle containing (0.1% DMSO in PBS) was used as control. MCC950 or corresponding vehicle was applied 1 h before the behavioral test. MCC950 was applied via lumbar catheterization as we described before [32]. Briefly, rats were anesthetized with sodium pentobarbital (50 mg/kg, i.p.). The lumbar part was shaved and disinfected using 75% alcohol beforehand. A small incision was cut along L4–5 lumbar vertebrae. The intervertebral foramen was exposed after cutting intervertebral ligament. A PE-10 catheter prefilled with sterilized PBS was inserted into the subarachnoid space. The proper insertion of the catheter was indicated by tail-flick or paw retraction. After the insertion, the proper intraspinal location was examined by lidocaine (1%, 10 μl/rat) injection via a catheter. The proper intraspinal location of the catheter was indicated by a quick motor paralysis of the hind limbs of the rat, usually lasting 15–30 min. The catheter was then secured and skin incision was sutured. The rats were placed back into individual cages for further recovery.

### Western blot

Rats were sacrificed after behavioral testing on days 7. Rats were deeply anesthetized using pentobarbital (50 mg/kg, i.p.) and transcardially perfused with 200 mL normal saline (4 °C). The lumbar spinal cord was immediately removed and stored at − 80 °C. Tissues were homogenized in RIPA buffer (50 mM Tris [pH 7.4], 150 mM NaCl, 1% Triton X-100, 1% sodium deoxycholate, sodium orthovanadate, 0.1% SDS, EDTA, sodium fluoride, leupeptin, and 1 nM PMSF) and then centrifuged at 15,000 rpm for 15 min at 4 °C and the supernatant was collected. The protein concentration was determined using the BCA method according to the kit’s instruction (Thermo Fisher, USA) and 20 μg protein was loaded in each lane. Protein samples were separated on 8–12% SDS-PAGE gels and electrophoretically transferred to polyvinyl difluoride (PVDF) membranes (Merck KGaA, Darmstadt, Germany). The membranes were blocked with 5% non-fat milk at room temperature for 1 h, followed by overnight incubation at 4 °C with the following primary antibodies diluted in blocking buffer: IL-1β (1:500, rabbit polyclonal, #ab9722, Abcam), NLRP3 (1:500, rabbit polyclonal, #NBP2-12446, Novus), Caspase-1 (1:500, rabbit polyclonal, ab1872, Abcam). Subsequently, the immunoblots were incubated with the 2nd antibodies for 1 h at room temperature. Mouse anti-β-actin (HRP Conjugate, 1:5000, #ab20272, Abcam) was used as reference control. The immunoreactivity was detected using enhanced chemiluminescence (BIO-RAD, USA) and visualized with an Image Quant LAS 4000 (EG, USA). The density of each band was measured using Image Quant TL 7.0 analysis software (GE, USA). The expression level of the target protein was normalized with β-actin protein expression level.

### Statistical analysis

Data in graphs are expressed as means ± SEM. One- or two-way ANOVA followed by Tukey’s post hoc test was used for comparison among groups ≥ 3. Student’s *t* test was used for comparisons between two groups. Comparison is considered significantly different if the *p* value < 0.05.

## Results

### CPIP model rats showed persistent mechanical allodynia and glial activation in the spinal cord dorsal horn

**Fig. 1:**
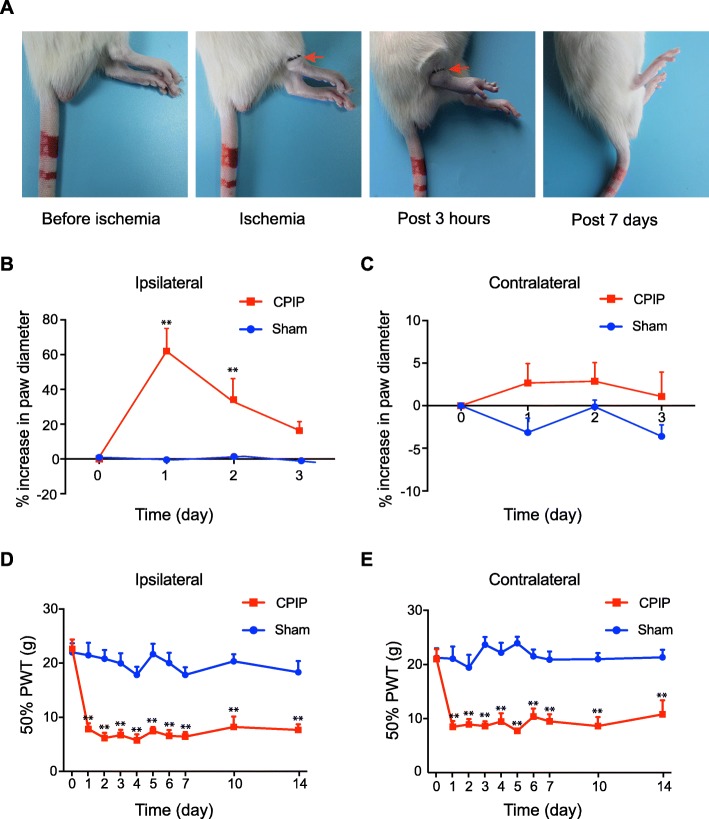
The chronic post-ischemia pain (CPIP) rat model exhibits persistent mechanical allodynia. **a** Representative photographs of rat hind paw taken before the CPIP model establishment, during ischemia, 3 h and 7 days after the model establishment. The red arrow indicates the O-ring clamped right above the ankle. **b**, **c** The paw swelling of ipsilateral and contralateral hind paws of CPIP and Sham group rats. **d**, **e** 50% paw withdraw threshold (PWT) of ipsilateral and contralateral hind paws of sham and CPIP group rats. **p < 0.01 *vs*. sham group. *n* = 8 rats/group. Two-way ANOVA followed by Tukey’s post hoc test was used for comparison

**Fig. 2:**
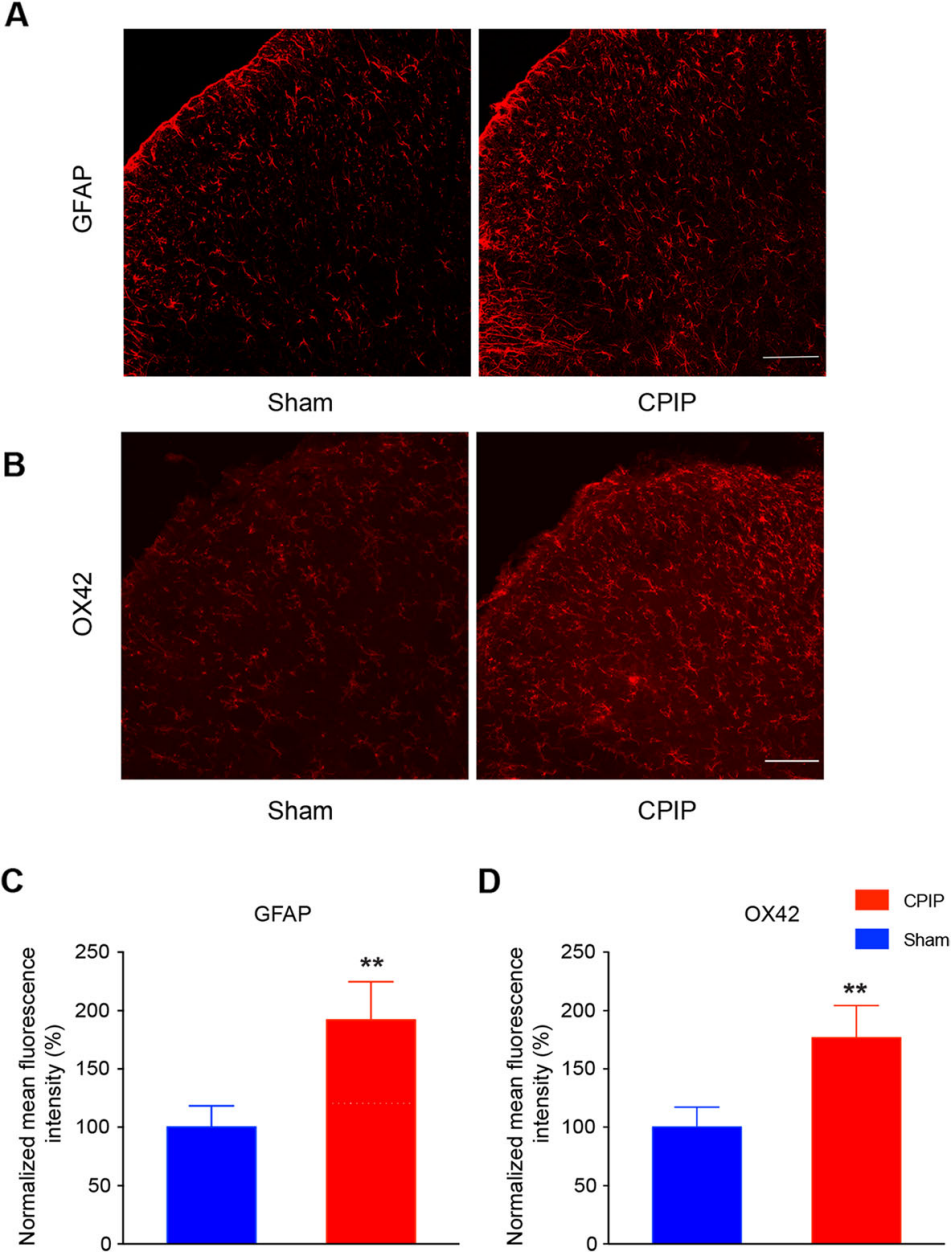
Astrocytes and microglia are activated in ipsilateral SCDH of CPIP model rats. **a** The expression of GFAP in ipsilateral SCDH of sham and CPIP group. **b** The expression of OX-42 in ipsilateral SCDH of sham and CPIP group. **c**, **d** The summarized normalized % increase in the mean fluorescence intensity of GFAP (**c**) and OX-42 staining (**d**). The value was normalized to sham group. Scale bar indicates 50 μm. **p < 0.01 vs. sham group. Student’s *t* test was used for comparisons

We first established the rat model of CPIP to mimic human CRPS-I according to methods described before [9]. As shown in Fig. 1a–c, the ipsilateral hind paw showed obvious edema compared with the contralateral hind paw 3 h after ischemia and returned normal 3 days later. In contrast, CPIP model rats displayed long-lasting (> 14 days) mechanical allodynia in both ipsilateral and contralateral hind paws (Fig. 1d, e). These signs are consistent with previous publications and suggested successful establishment of the CPIP model [9]. We sacrificed the rats and collected the spinal cord dorsal horn at day 7 and examined the expression pattern of GFAP and OX-42, markers for astrocyte and microglia, respectively. As shown in Fig. 2 a-d, the expression of GFAP and OX-42 were both significantly increased in the ipsilateral SCDH of CPIP model rats compared with sham rats, an indication of glial cell activation in SCDH of CPIP model.

### Examining gene expression profiles of SCDH after CPIP by RNA-Seq

**Fig. 3:**
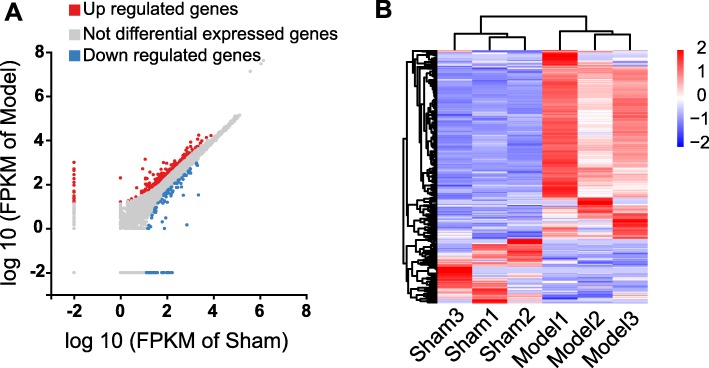
RNA-Seq reveals gene expression changes in SCDH induced by CPIP. **a** Scatter plot showing gene expression profiles in ipsilateral SCDH of CPIP group compared with sham group. Red and blue spots indicate up- and downregulated DEGs, respectively, whereas gray spots indicate non-DEGs. **b** Heat map displaying the hierarchical clustering of DEGs from CPIP and sham group

In order to explore the genes that possibly participate in pain mechanisms of CPIP model, we harvested L4-6 SCDH of CPIP model and sham rats and analyzed the gene expression profiles via RNA-Seq. The RNA-Seq generated 21.94 million raw reads per sample and the clean reads Q20% reached nearly 99.0% (Table 1). More than 90% of bases reached a quality score ≥ Q30 and over 95% of the clean reads data were mapped to the rat genome (Table 1). Eventually, 18,672 genes were successfully mapped and identified from RNA-Seq (Additional file 1: Suppl. Table 1). We then set to identify differentially expressed genes (DEGs) with criteria of fold change ≥ 2 and *q* value ≤ 0.01. Based upon this criterion, we identified a total of 435 DEGs (including 324 up- and 111 downregulated), which are further displayed in the scatter plot graph (Fig. 3a, Additional file 2: Suppl. Table 2). The identified DEGs were then summarized and plotted in the heat map (Fig. 3b). Cluster analysis indicated a high level of concordance within both CPIP and Sham group samples and a clear segregation between the two groups (Fig. 3b).

### Analysis of DEGs in SCDH of CPIP model rats

Among the DEGs that we identified, some of the genes are known to be related with inflammation or pain processes, such as *Itgam* (integrin subunit alpha M, fold change = 261.4), *Cxcl13* (C-X-C motif chemokine 13 precursor, fold change = 54.9), *C3* (complement component 3, fold change = 7.1), *Cd68* (Cd68 molecule, fold change = 5.2), and *Il-1β,* (interleukin-1 beta, fold change = 3.8). We further analyzed the data and found that 77 genes showed expression changes more than 10-fold, with 31 upregulated genes and 46 downregulated gene, such as *Isoc1* (isochorismatase domain-containing protein 1, fold change = 845.7), *Stra8* (stimulated by retinoic acid 8, fold change = 192.4), *Rpl30* (ribosomal protein L30, fold change = 115.7); 33 genes showed expression changes between 5- and 10-fold, which include 24 upregulated and 9 downregulated genes. The detailed information of the top 20 up- and 20 downregulated DEGs are summarized in Tables 3 and 4, respectively.

**Table 3:** The detailed information of the top 20 upregulated DEGs

Upregulated gene	Gene ID	Location	Log2 fold change (CPIP/Sham)	*Q* value	Official gene name (NCBI)
*LOC100363502*	100363502	62475574–62476771	10.9252125	9.77E− 139	
*LOC103689943*	103689943	28267–72912	9.9331474	2.03E− 81	
*Isoc1*	364879	53727203–53747612	9.72392644	1.35E− 72	Isochorismatase domain-containing protein 1
*LOC100911615*	100911615	213997721–214002811	9.07094965	1.79E− 50	
*LOC100911825*	100911825	91219838–91237274	8.61974834	2.04E− 24	
*LOC685963*	685963	164002409–164002748	8.46420752	9.52E− 36	
*LOC103689965*	103689965	4495440–4508232	7.92974175	3.41E− 26	
*Stra8*	500079	62447724–62474802	7.58808827	2.55E− 42	Stimulated by retinoic acid 8
*LOC100909795*	100909795	78550661–78577178	7.48236683	4.25E− 20	
*Rpl30*	64640	73213538–73216431	6.85524869	0	Ribosomal protein L30
*LOC108348184*	108348184	1622570–1683919	6.59189138	9.95E− 12	
*LOC103690173*	103690173	3738–18580	6.44436116	1.03E− 10	
*LOC103694874*	103694874	23060–31871	5.87292704	1.62E− 07	
*Cxcl13*	498335	15253146–15258221	5.78195338	1.23E− 117	C-X-C motif chemokine ligand 13
*Rsph4a*	309767	32450701–32467362	5.59130694	2.57E− 06	Radial spoke head component 4A
*LOC100912393*	100912393	3940948–3965964	5.45532247	8.21E− 06	
*Rps20*	122772	16706052–16707214	5.41559782	1.14E− 05	Ribosomal protein S20
*Batf*	299206	109562415–109584541	5.13596145	8.93E− 05	Basic leucine zipper ATF-like transcription factor
*Aqp3*	65133	57423735–57429252	5.13596145	8.93E− 05	Aquaporin-3
*Crkl*	287942	87338606–87356644	4.94331637	0.00030049	Crk-like proto-oncogene, adaptor protein

**Table 4:** The detailed information of the top 20 downregulated DEGs

Downregulated gene	Gene ID	Location	Log2 fold change (CPIP/Sham)	*Q* value	Official gene name (NCBI)
*Rps6*	29304	105197821–105200681	− 8.9793272	3.56E− 136	Ribosomal protein S6
*LOC102549712*	102549712	48508746–48542941	− 8.4277401	3.35E− 34	
*LOC100910581*	100910581	21987993–21989200	− 8.0682673	9.43E− 28	
*LOC100910446*	100910446	23542376–23560911	− 7.9258687	1.52E− 25	
*Rpl9*	29257	44524419–44527613	− 7.9088834	2.73E− 25	Ribosomal protein L9
*Triap1*	108348066	13511349–13513661	− 7.8860873	5.88E− 25	TP53-regulated inhibitor of apoptosis 1
*Pddc1*	309110	214389006–214394451	− 7.6510269	1.02E− 21	Glutamine amidotransferase-like class 1 domain containing 1
*Slc40a1*	108348131	52894300–52913267	− 7.2422467	5.11E− 17	Solute carrier family 40 member 1
*LOC688583*	688583	51458436–51462755	− 7.2391214	5.50E− 17	
*LOC102550456*	102550456	21748608–21761500	− 7.1733103	2.53E− 16	
*Ccnj*	294053	259926537–259944275	− 7.0687075	2.52E− 15	Cyclin J
*Snip1*	108348099	143063206–143070899	− 7.0547769	3.38E− 15	Smad nuclear interacting protein 1
*LOC108348298*	108348298	4487809–4499727	− 6.9606747	2.37E− 14	
*LOC108349606*	108349606	166282044–166282845	− 6.9454155	3.21E− 14	
*Scgb1a1*	25575	225279698–225283246	− 6.3193658	9.30E− 19	Secretoglobin family 1A member
*Rpl35a*	57809	7186459–7186874	− 6.3178853	1.09E− 09	Ribosomal protein L35a
*LOC100360679*	100360679	104521700–104522246	− 6.1465009	0	
*LOC103689971*	103689971	35396229–35518693	− 6.0905471	2.02E− 08	
*Prss1*	24691	70776046–70779249	− 5.9881599	6.66E− 08	Serine protease 1
*Sftpa1*	24773	18716019–18719404	− 5.7888511	5.52E− 07	Surfactant protein A1

### RNA-Seq data validation using qPCR

**Fig. 4:**
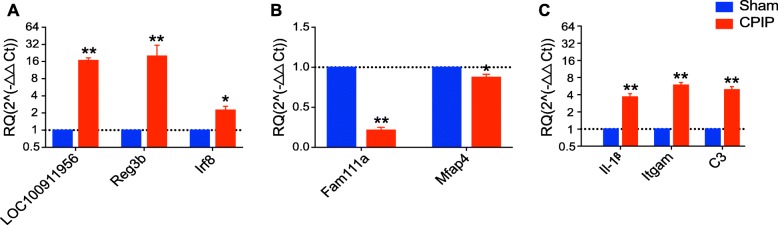
The validation of RNA-Seq results via qPCR. **a** The expression of three randomly selected upregulated DEGs from RNA-Seq was examined by qPCR. **b** The expression of two randomly selected downregulated DEGs from RNA-Seq was examined by qPCR. **c** The expression of some typical genes involved in inflammation and pain was examined by qPCR. *n* = 6 rats/group. *p < 0.05, **p < 0.01 vs. sham group. Student’s *t* test was used for comparisons

Next, we set to examine the reliability of our RNA-Seq data using qPCR. We randomly selected three upregulated genes (*LOC100911956*, *Reg3b*, and *Irf8*) and two downregulated genes (*Fam111a* and *Mfap4*) from the DEGs list and verified their expression via qPCR. The results of qPCR showed that the expression of *LOC100911956*, *Reg3b*, and *Irf8* were all significantly upregulated, whereas *Fam111a* and *Mfap4* were both significantly downregulated in ipsilateral SCDH of CPIP model rats *vs*. sham rats (Fig. 4a, b), which are consistent with RNA-Seq data. Furthermore, we continued to examine some representative genes which are well documented to participate in neuroinflammation or pain mechanisms, including *Il1β, Itgam,* and *C3*, by qPCR. The qPCR data suggested that the expression of *Il1β*, *Itgam,* and *C3* were all significantly upregulated in SCDH of CPIP model rats *vs*. sham rats (Fig. 4c), consistent with the RNA-Seq data. Thus, the results of qPCR provide evidence showing that the RNA-Seq data for gene expression profiling was reliable.

### Function and pathway analysis of DEGs in SCDH of CPIP model rats

**Fig. 5:**
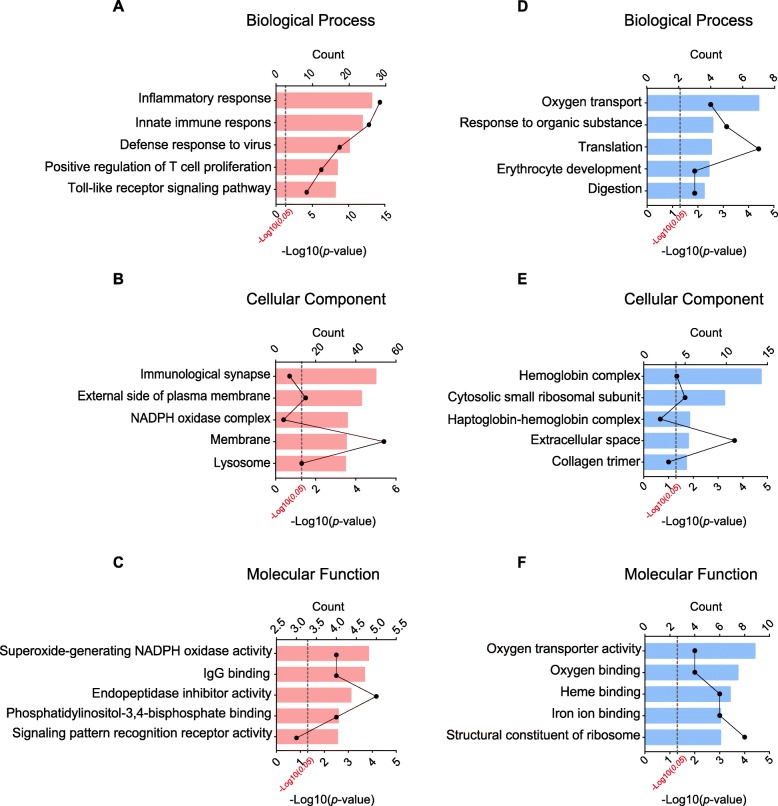
GO pathway analysis of DEGs. **a**–**c** The top 5 significant biological processes, cellular components, and molecular functions of upregulated DEGs. **d**–**f** The top 5 significant biological processes, cellular components, and molecular functions of downregulated DEGs. The dotted line indicates the *p* value of 0.05

In order to further explore the mechanisms underlying CRPS-I, we carried out gene ontology (GO) analysis of the DEGs in SCDH of CPIP and sham group of rats. According to functional annotation in GO database, we found that the most significantly enriched biological process of upregulated DEGs in CPIP model group were inflammatory response, innate immune response, defense response to virus, etc. (Fig. 5a, Additional file 3: Suppl. Table 3). The most significantly enriched cellular component of upregulated DEGs in CPIP model group was immunological synapse, external side of plasma membrane and NADPH oxidase complex, etc. (Fig. 5b, Additional file 4: Suppl. Table 4). The most significantly enriched molecular function of upregulated DEGs in CPIP model group was superoxide-generating NADPH oxidase activity, IgG binding, endopeptidase inhibitor activity, etc. (Fig. 5c, Additional file 5: Suppl. Table 5). Meanwhile, the most significantly enriched biological process of downregulated DEGs in CPIP model group was oxygen transport, response to organic substance, translation, etc. (Fig. 5d, Additional file 6: Suppl. Table 6). The most significantly enriched cellular component of downregulated DEGs in CPIP model group was hemoglobin complex, cytosolic small ribosomal subunit and haptoglobin-hemoglobin complex, etc. (Fig. 5e, Additional file 7: Suppl. Table 7). The most significantly enriched molecular function of downregulated DEGs in CPIP model group was oxygen transporter activity, oxygen binding and heme binding, etc. (Fig. 5f, Additional file 8: Suppl. Table 8).

**Fig. 6:**
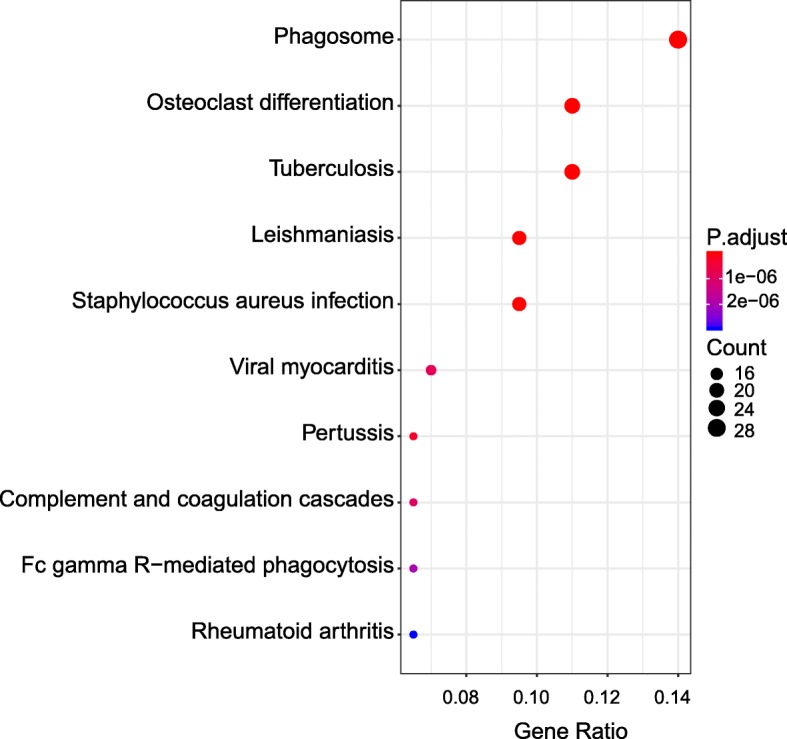
KEGG pathway analysis of DEGs. Bubble plots showing the significant pathways for up- and downregulated DEGs. Larger bubbles indicate higher number of genes. The color of each bubble reflects significance (*p* value)

We further performed KEGG pathway enrichment analysis of the DEGs between CPIP model and sham groups. Scatter diagrams of KEGG measured the degree of enrichment by rich factor, *q* value and gene number of the enriched pathway. Based upon the KEGG pathway enrichment analysis, we found that the DEGs in SCDH of CPIP model group were mainly related with phagosome, osteoclast differentiation, tuberculosis, complement and coagulation cascades and phagocytosis, etc. (Fig. 6, Additional file 9: Suppl. Table 9).

### Comparison of RNA-Seq dataset of CPIP model with other neuropathic pain model datasets

**Fig. 7:**
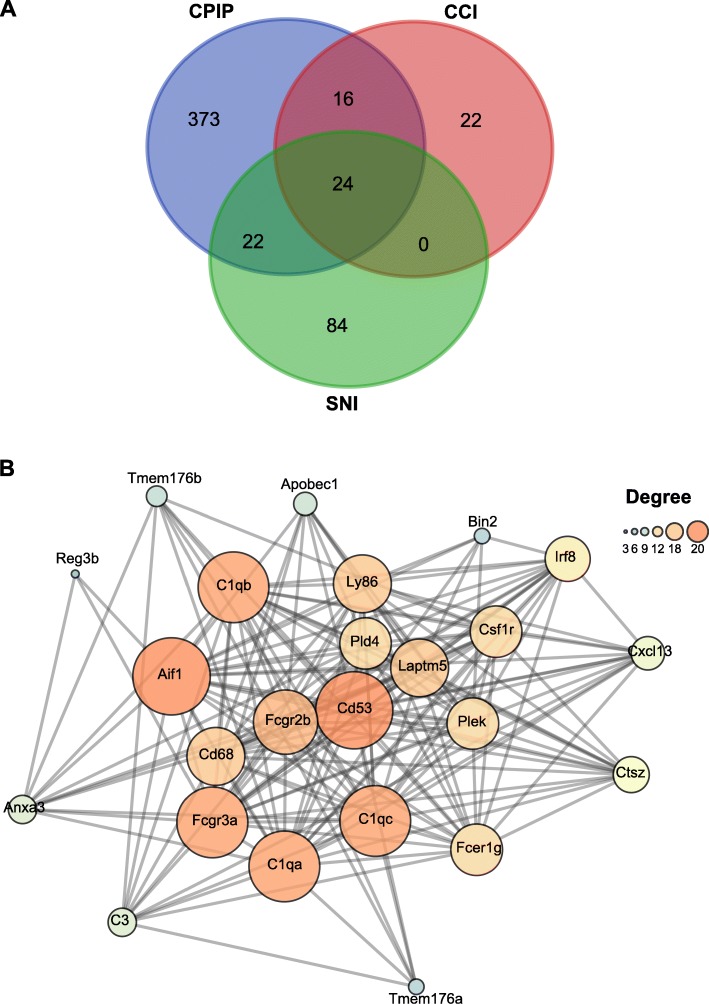
Comparison of our present CPIP RNA-Seq datasets with other published microarray/RNA-Seq datasets of neuropathic models. **a** Venn diagram showing the overlapping of DEGs of SCDH from CPIP rat model with rat SNI and CCI neuropathic pain models. **b** PPI network analysis of the DEGs overlapped with SNI and CCI gene datasets. Larger circles and darker colors indicate higher degree of interactions

In our previous study, we compared DEGs of DRGs from CPIP model rats with other well-established neuropathic or inflammatory pain models and found that CPIP model exhibited more changes in neuropathic-pain-related genes than inflammatory-pain-related genes [13]. In the present study, we continued to compare our RNA-Seq data derived from SCDH of CPIP model rats with the datasets obtained from two other well-established rat models of neuropathic pain, namely, the SNI and CCI models. Two gene profiling datasets of CCI and SNI models were downloaded as described in the “Materials and methods” section. We imposed the same criteria (fold change ≥ 2 or ≤ − 2, *q* ≤ 0.01) upon both SNI and CCI datasets for identifying DEGs. We found that the DEGs of CPIP model group had 46 and 40 genes overlapping with the DEGs of SNI and CCI model, respectively, accounting for 10.6% and 9.2% of all DEGs of CPIP model (Fig. 7a, Additional file 10: Suppl. Table 10, Additional file 11: Suppl. Table 11). Besides, the three groups shared a core set of 24 genes that were overlapped together (Fig. 7a, Table 5). These 24 genes all showed upregulation in the SCDH of CPIP model group. Finally, in order to study the interactions and the hub genes of DEGs that are involved in CPIP and other neuropathic pain models, we carried out PPI network analysis of these 24 genes and found that the major hub genes deduced from PPI analysis include *Cd53, Aif1, C1qc, C1qa, C1qb, Fcgr3a,* and *Fcgr2b* (Fig. 7b).

**Table 5:** The 24 DEGs of CPIP overlapping with the SNI and CCI datasets

Genes symbol	Official gene name(NCBI)	Change in three datasets
Cxcl13	C-X-C motif chemokine ligand 13	Up
C3	Complement component 3	Up
Reg3b	Regenerating family member 3 beta	Up
C1qc	Complement C1q C chain	Up
Cd68	Cd68 molecule	Up
C1qa	Complement C1q A chain	Up
C1qb	Complement C1q B chain	Up
Fcgr3a	Fc fragment of IgG receptor IIIa	Up
Apobec1	Apolipoprotein B mRNA editing enzyme catalytic subunit 1	Up
Irf8	Interferon regulatory factor 8	Up
Fcgr2b	Fc fragment of IgG receptor IIb	Up
Pld4	Phospholipase D family, member 4	Up
Bin2	Bridging integrator 2	Up
Ly86	Lymphocyte antigen 86	Up
Ctsz	Cathepsin Z	Up
Fcer1g	Fc fragment of IgE receptor Ig	Up
Anxa3	Annexin A3	Up
Tmem176a	Transmembrane protein 176A	Up
Csf1r	Colony stimulating factor 1 receptor	Up
Aif1	Allograft inflammatory factor 1	Up
Tmem176b	Transmembrane protein 176B	Up
Plek	Pleckstrin	Up
Cd53	Cd53 molecule	Up
Laptm5	Lysosomal protein transmembrane 5	Up

In order to explore which cell type in SCDH is particularly affected by CPIP model, we performed gene set enrichment analysis (GSEA) using gene sets well-established for astrocyte activation (systematic name, M23832), microglia activation (M25223), and oligodendrocyte differentiation (M15768) from the Molecular Signatures Database v7.1. We found that the CPIP model showed significant activation of the three gene sets by GSEA analysis (NES > 1.0, FDR ≤ 0.25), indicating that all three cell types in the SCDH are affected by CPIP model (Suppl. Fig. 1A-C). As a comparison, we also performed GSEA analysis of the SNI neuropathic pain model, in which the activation of astrocytes and microglia in SCDH are well established. As a result, GSEA analysis revealed that SNI model showed similar activation of the three gene sets compared with CPIP model (Suppl. Fig. 1D-F). Therefore, GSEA analysis suggests that the microglia, astrocytes, and oligodendrocytes in SCDH are all significantly affected in CPIP model.

### Identifying NLRP3 inflammasome in SCDH as a key player in mediating the pain and inflammation responses in CPIP rat model.

**Fig. 8:**
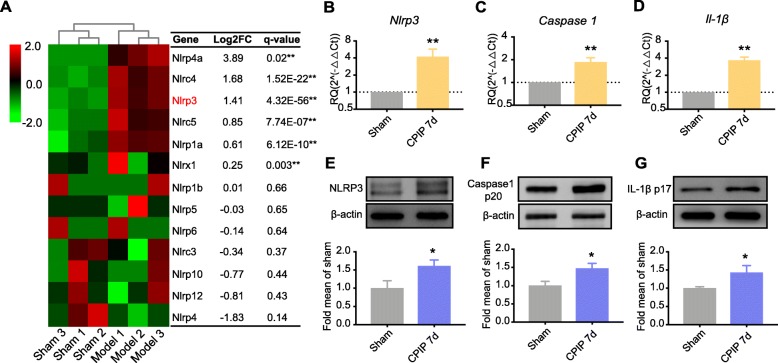
NLRP3 inflammasome is identified to be significantly upregulated in ipsilateral SCDH of CPIP rats. **a** Heat map showing the expression of NLR family genes identified in ipsilateral SCDH of CPIP rats vs. sham rats. *n* = 3 rats/group. **b**–**d** qPCR validation of the upregulation of *Nlrp3* (**b**), *Caspase-1* (**c**), and *Il-1β* (**d**) genes in ipsilateral SCDH of CPIP rats vs. sham rats. *n* = 8 rats/group. **e**–**g** Western blot analysis of NLRP3 (**e**), Caspase-1 (**f**), and IL-1β (**g**) protein expressions in ipsilateral SCDH of CPIP rats vs. sham rats. *n* = 8 rats/group. *p < 0.05, **p < 0.01 vs. sham group. Student’s *t* test was used for comparisons

We observed *Il-1β* gene is significantly upregulated in SCDH of CPIP rats (Fig. 4c). IL-1β overproduction in SCDH has recently been shown to be important for the neuroinflammation and pain response of a mouse model of CRPS-I [33]. However, the precise mechanisms of how IL-1β is overproduced under CRPS-I is still unknown. NLRP3 inflammasome is a complex of proteins responsible for the cleavage of pro-IL-1β into active IL-1β, which plays an important role in mediating pain response, inflammation, and central sensitization [34–37]. We therefore analyzed all NLR family gene changes in SCDH of CPIP model rats vs. sham rats and found that *Nlrp3* gene is significantly upregulated (Fig. 8a). In contrast, *Nlrp3* gene expression is not significantly altered in CCI or SNI model rats by analyzing the dataset as mentioned in Fig. 7, suggesting *Nlrp3* gene expression upregulation in SCDH is specific to CPIP model rats. We therefore continued to validate the *Nlrp3* gene expression by qPCR. qPCR showed that the mRNA expression of the three major components in NLRP3 inflammasome, namely *Nlpr3*, *Caspase-1,* and *Il-1β*, are all significantly upregulated in ipsilateral SCDH of CPIP model rats 7 days after model establishment (Fig. 8b–d). Moreover, Western blot studies further confirmed that the protein expressions of NLRP3, Caspase-1, and IL-1β are all significantly upregulated in ipsilateral SCDH of CPIP model rats 7 days after model establishment (Fig. 8e–g), suggesting the activation of NLRP3 inflammasome in SCDH of CPIP model rats.

**Fig. 9:**
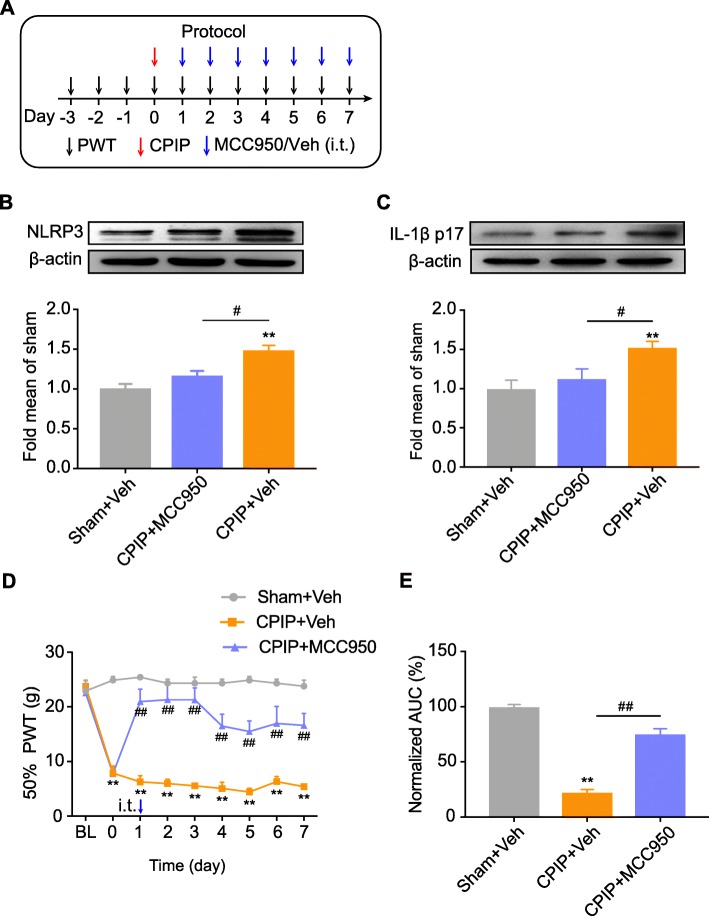
Pharmacological blocking NLRP3 inflammasome activation attenuated the mechanical allodynia of CPIP rats. **a** Schematic protocol illustrating the time points for model establishment, behavioral tests, and MCC950 (30 μg/rat in 12.5 μl injection volume, via intrathecal catheter)/vehicle (0.1% DMSO in PBS) application. **b** NLRP3 expression in ipsilateral SCDH of Sham+Veh, CPIP+MCC950, and CPIP+Veh groups measure by Western blot. Upper panel indicates representative images of NLRP3 and β-actin protein expression. Lower panel indicates summarized NLRP3 expression normalized to β-actin. **c** IL-1β expression in ipsilateral SCDH of Sham+Veh, CPIP+MCC950, and CPIP+Veh groups measure by Western blot. Upper panel indicates representative images of IL-1β and β-actin protein expression. Lower panel indicates summarized IL-1β expression normalized to β-actin. *n* = 4 rats/group. **d** Time course effect of MCC950 on 50% paw withdraw threshold (PWT) of ipsilateral hind paw of CPIP rats. **e** Summary of the normalized area under the curve (AUC) as in **d**. *n* = 6 rats/group. **p < 0.01 vs. Sham+Veh group. ^##^*p* < 0.01 vs. CPIP+Veh group. Two-way ANOVA followed by Tukey’s post hoc test was used for comparison in panel **d**. One-way ANOVA followed by Tukey’s post hoc test was used for comparison in panel **e**

**Fig. 10:**
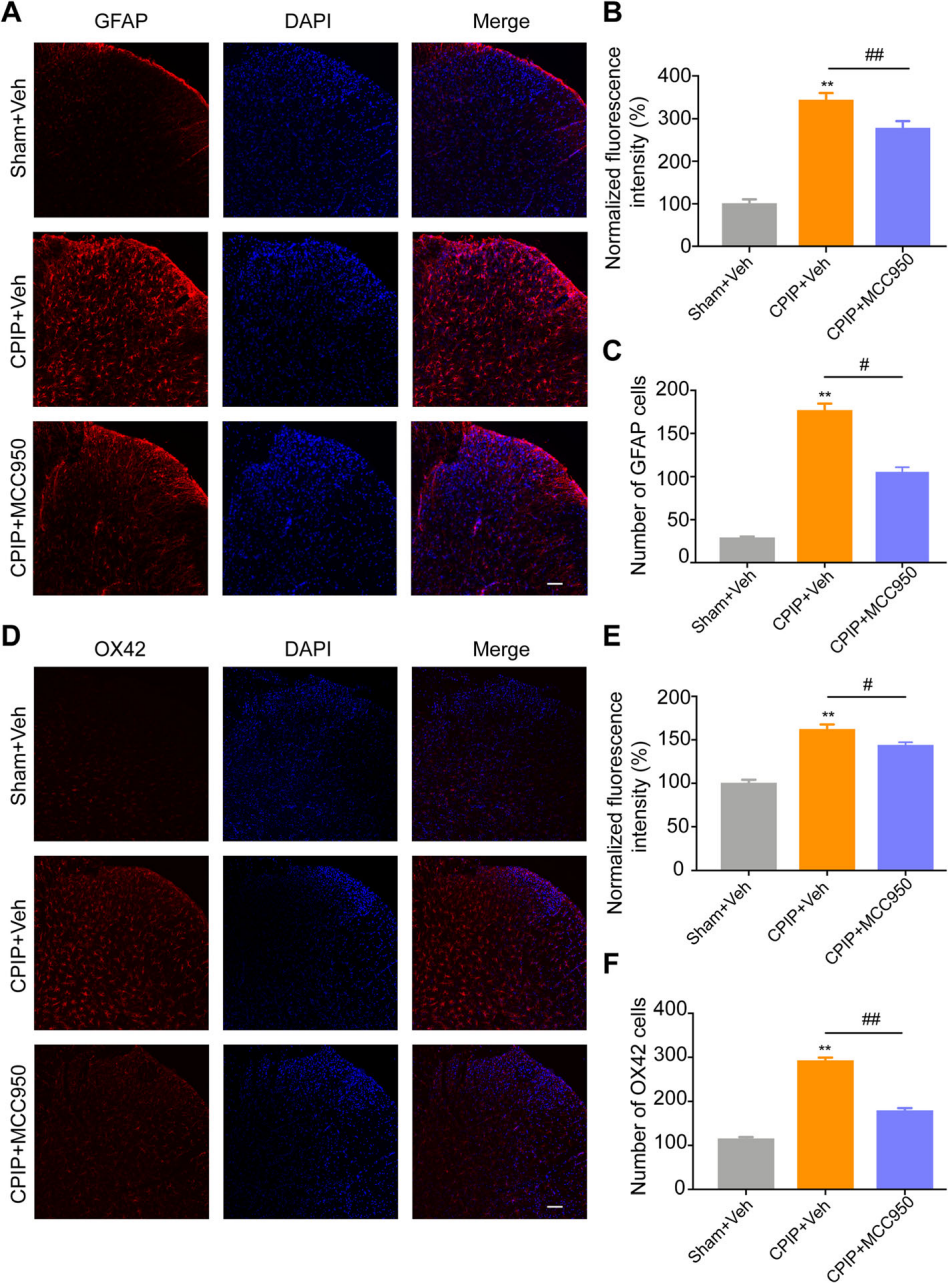
Pharmacological blocking NLRP3 inflammasome activation attenuates astrocyte and microglial activation in SCDH of CPIP rats. **a** Representative photos of ipsilateral SCDH stained with GFAP antibody showing astrocytes from Sham+Veh, CPIP+Veh, and CPIP+MCC950 group. Scale bar = 100 μm. **b** Summary of the normalized fluorescence intensity (%) of GFAP staining in ipsilateral SCDH. *n* = 5 rats/group. **c** Summary of the number of astrocytes observed in ipsilateral SCDH per observation field. *n* = 5 rats/group. **d** Representative photos of ipsilateral SCDH stained with OX42 antibody showing microglia from Sham+Veh, CPIP+Veh, and CPIP+MCC950 group. Scale bar = 100 μm. **e** Summary of the normalized fluorescence intensity (%) of OX42 staining in ipsilateral SCDH. *n* = 6 rats/group. **f** Summary of the number of microglia observed in ipsilateral SCDH per observation field. *n* = 6 rats/group. ^**^p < 0.01 *vs*. Sham+Veh group. ^#^*p* < 0.05, ^##^*p* < 0.01 vs. CPIP+Veh group. One-way ANOVA followed by Tukey’s post hoc test was used for comparison

We next examined whether NLRP3 inflammasome contributed to the pain response of CPIP model rats. In order to address this issue, we used MCC950, a potent and specific inhibitor of NLRP3 inflammasome [38, 39]. MCC950 or corresponding vehicle (0.1% DMSO in PBS) was applied through intrathecal catheter on a daily basis after model establishment (Fig. 9a). As expected, intrathecal MCC950 application significantly reduced the upregulation of NLRP3 and IL-1β overexpression in ipsilateral SCDH of CPIP model rats (Fig. 9b, c). More importantly, intrathecal MCC950 significantly attenuated the mechanical allodynia of ipsilateral hind limbs of CPIP model rats vs. CPIP model rats injected with vehicle only (Fig. 9d). Area under the curve (AUC) analysis further indicated an overall attenuation of the mechanical allodynia by accumulated MCC950 treatment (Fig. 9e). To further study the cellular mechanisms underlying NLRP3 inflammasome in mediating the pain response of CPIP model rats, we investigated whether NLRP3 inflammasome participates in the activation of spinal astrocytes and microglia of CPIP model rats, which are crucial steps involved in central pain transduction and sensitization [40, 41]. Immunostaining found that the overexpression of GFAP or OX42 in ipsilateral SCDH of CPIP model rats was significantly reduced by intrathecal MCC950 treatment (Fig. 10a–f). Thus, the above data indicated that NLRP3 inflammasome contributes to the mechanical allodynia of CPIP model rats via mechanisms possibly involving activation of astrocytes and microglial in SCDH.

## Discussion

In the present study, we examined the gene expression profiles in ipsilateral L4-6 SCDH of the CPIP model and sham rats through RNA-Seq. We identified a number of DEGs and validated their expressions via qPCR. We further looked into the molecular and cellular functions of these DEGs by applying GO and KEGG analysis and found that the most significantly enriched biological processes of upregulated genes are involved in inflammatory response and innate immune response. We further compared our dataset with other published datasets of common neuropathic pain models and identified a core set of genes and pathways that extensively participate in CPIP and other neuropathic pain models. To our knowledge, this is by far the first study to profile the gene expression changes and key pathways in the SCDH of a rat model of CRPS-I using RNA-Seq.

Previous studies have demonstrated that central sensitization plays a critical role in maintaining chronic pain [42]. There is increasing evidence that central sensitization is driven by neuroinflammation of the surrounding and central nervous system [43]. A key feature of neuroinflammation is the activation of glial cells, such as microglia and astrocytes in the spinal cord and brain, which results in the release of an array of pro-inflammatory cytokines and chemokines that could sensitize pain-processing neurons in the spinal dorsal horn [44, 45]. In this study, we found that CPIP model rats showed significant activation of microglia and astrocytes in the ipsilateral SCDH, which is consistent with previous studies and our recent findings [11, 14]. The persistent microglia and astrocytes activation in the SCDH leads to sustained release of pro-inflammatory cytokines and chemokines in the spinal cord, which contributes to chronic pain condition manifested in CPIP model rats [11]. Therefore, in the present study, we performed the expression profiling of the SCDH of CPIP model rats, aiming to unravel the molecular mechanisms underlying CRPS-I.

GO analyses revealed that the most significantly enriched biological processes of upregulated genes in SCDH of CPIP vs. sham group were related with inflammatory and innate immune response, defense response to virus, positive regulation of T cell proliferation and Toll-like receptor signaling pathway, etc. This result implies that neuroinflammation in SCDH may constitute a predominant mechanism involved in the pathophysiology of the CRPS-I. Besides, GO analysis of molecular function revealed that superoxide-generating NADPH oxidase activity is among the most significantly enriched function in the SCDH of CPIP model rats. NADPH oxidases are the main enzymes that produce reactive oxygen species (ROS) and trigger subsequent oxidative stress. NADPH oxidase-mediated ROS production and oxidative stress are involved in persistent pain, including neuropathic and inflammatory pain [46, 47]. Reducing ROS generation alleviates pain in CPIP model rats [48, 49]. Therefore, the above results suggest that targeting neuroinflammation and NADPH oxidase-mediated ROS production may be potential effective strategies to ameliorate CRPS-I.

The KEGG analysis unraveled that the most enriched pathway the DEGs are involved in is related with phagosome. This result is consistent with our recent findings that phagosome is a predominant process that took place in the peripheral DRGs of CPIP model rats [13]. Similar result was also observed in another study performing RNA-Seq on SCDH of CCI-induced neuropathic pain model [29]. One of the representative genes involved in phagosome is *Itgam*, a marker for macrophage and microglia. In our study, *Itgam* gene upregulation is detected by both RNA-Seq and qPCR. We also found that microglia is significantly accumulated in the ipsilateral SCDH of CPIP model rat, a result consistent with other studies [11, 15]. Accumulated microglia cells release BDNF and increase neuronal excitability in SCDH by via neuron-glial crosstalk, which contributes to pain in CPIP [11]. Therefore, our data, together with the others, suggest that microglia in the SCDH may play an important role in mediating pain symptom and neuroinflammation of CRPS-I.

In the present study, cluster analysis indicated a high level of concordance of datasets within both CPIP and sham group samples and a clear segregation between the two groups. However, we noticed that some gene expression changes in model 1 are more robust than model 2 and 3, which possibly means that some of the gene changes may be driven by a single sample in the CPIP model. Since only 3 biological samples are included in each group, therefore, we have to acknowledge that our present RNA-Seq dataset may possess certain weakness due to the limited number of samples included. However, it should be noted that RNA-Seq is a preliminary data screening method. qPCR and protein assay should be followed for further validation of targets. In our study, we carried out further qPCR and Western blot experiments using different batches of animals to validate our RNA-Seq data and we successfully identified many genes and targets that showed a consistent trend with our RNA-Seq dataset. Therefore, we hope future studies which incorporate more samples into RNA-Seq analysis could be carried out and compare with our present data.

NLRP3 inflammasome consists of a complex of proteins, including NLRP3/ASC/caspase-1, which are responsible for the cleavage of pro-IL-1β into active IL-1β [50, 51]. IL-1β has been well known as a potent pain mediator which sensitizes or directly activates the nociceptors [52]. It has been reported that peripheral NLRP3 inflammasome activation in the peripheral sensory nerves or tissues contributes to paclitaxel-induced neuropathic pain or inflammatory pain [34, 35]. However, the exact contribution of NLRP3 inflammasome in the SCDH to pain mechanisms is still not fully understood yet. Here, we successfully identified the genes encoding the NLRP3 inflammasome complex, including *Nlrp3*, *Caspase-1,* and *Il-1β*, to be significantly upregulated in SCDH of CPIP rats by RNA-Seq and qPCR. Western blot further validated our results, showing that the protein expression of NLRP3, Caspase-1, and IL-1β are all upregulated in SCDH of CPIP rats vs. sham rats. IL-1β is a well-established pro-inflammatory cytokine that can activate astrocytes and microglia in SCDH to produce mechanical allodynia [53, 54]. More recently, it was demonstrated that blocking IL-1β with neutralizing antibody prevented glial activation in SCDH and reduced pain response in a mouse CRPS-I model, suggesting an important role of IL-1β in the pathogenesis of CRPS-I [33]. We found that pharmacological blocking NLRP3 inflammasome activation by intrathecal MCC950 significantly reduced IL-1β overproduction in SCDH of CPIP rats. This indicates NLRP3 inflammasome is the predominant molecular source for IL-1β overproduction in CPIP condition. MCC950 treatment further attenuated activation of astrocytes and microglia in SCDH of CPIP rats and reduced the mechanical allodynia. Therefore, these results suggest that NLRP3 inflammasome may contribute to the mechanical allodynia of CPIP rats via mechanisms possibly involving IL-1β overproduction and activation of astrocytes and microglial in SCDH.

At present, male rats were used exclusively for CPIP model establishment in accordance with previous studies [9]. But epidemiological studies indicated that CRPS-I occurs more frequently in female than in male patients [55, 56]. This clinical finding is further supported by the observation that female animals showed more nocifensive responses than male animals of the CPIP model [57]. Therefore, it would be important to include both male and female animals in the CPIP model for future studies. This will be important for studying the translational significance of this CRPS-I animal model.

CRPS-I remains an enigmatic symptom that usually initiates after limb injury, fracture, or surgery [58]. The CPIP rat model we used in the present study mimics local tissue ischemia, which could result from limb injury, fracture, or surgery, to recapitulate human CRPS-I. Although it well recapitulates many clinical manifestations of CRPS-I in humans, the CPIP rat model still has its own limitation that it does not mimic the typical clinical factors that may trigger CRPS-I, such as limb injury, fracture, or surgery. More recently, it is proposed that CRPS is a post-traumatic autoimmune disease [58]. Therefore, some other animal models, such as the mouse tibia fracture model and the newly developed translational passive transfer trauma mouse model of CRPS may be used for further validation of our current findings [33, 59, 60].

## Conclusions

In conclusion, we presented the gene expression profiling data of ipsilateral SCDH of a rat model of CRPS-I using RNA-Seq. We uncovered a number of DEGs and pathways in the SCDH that may potentially participate in mediating the neuroinflammation and pain of CRPS-I. We further identified NLRP3 inflammasome in SCDH as a key contributor to the pain and inflammation responses in CPIP rats. These results may provide us insights into understanding the molecular mechanisms of CRPS-I and further help to develop novel and effective therapeutics against CRPS-I.

## Supplementary information

**Additional file 1: Suppl. Table 1.** All expressed transcripts.

**Additional file 2: Suppl. Table 2.** DEGs.

**Additional file 3: Suppl. Table 3.** BP result of the upregulated DEGs in CPIP.

**Additional file 4: Suppl. Table 4.** BP result of the downregulated DEGs in CPIP.

**Additional file 5: Suppl. Table 5.** CC result of the upregulated DEGs in CPIP.

**Additional file 6: Suppl. Table 6.** CC result of the downregulated DEGs in CPIP.

**Additional file 7: Suppl. Table 7.** MF result of the upregulated DEGs in CPIP.

**Additional file 8: Suppl. Table 8.** MF result of the downregulated DEGs in CPIP.

**Additional file 9: Suppl. Table 9.** KEGG analysis.

**Additional file 10: Suppl. Table 10.** Overlapping with SNI.

**Additional file 11: Suppl. Table 11.** Overlapping with CCI.

**Additional file 12: Suppl. Fig. 1.** Analysis of astrocyte activation, microglia activation and oligodendrocyte differentiation using GSEA. (A-C) GSEA analysis of CPIP model RNA-Seq dataset using well-defined gene sets for astrocyte activation, microglia activation and oligodendrocyte differentiation from the Molecular Signatures Database v7.1. (D-F) GSEA analysis of the SNI model dataset (GSE18803) for comparison with the CPIP model.

## Data Availability

The key data are contained in the figures, tables, and additional files. The datasets used and/or analyzed during this study can be obtained from the corresponding author on reasonable request.

## Abbreviations

CRPS-I: Complex regional pain syndrome type-I
CPIP: Chronic post-ischemic pain
RNA-Seq: RNA-sequencing
SCDH: Spinal cord dorsal horn
DEGs: Differentially expressed genes
NSAIDs: Non-steroidal anti-inflammatory drugs
DRG: Dorsal root ganglia
PWT: Paw withdrawal threshold
QC: Quality control
KEGG: Kyoto Encyclopedia of Genes and Genomes
DAVID: Data-base for Annotation, Visualization and Integrated Discovery
qPCR: Real-time quantitative PCR
SNI: Spared nerve injury
CCI: Chronic constriction injury
GEO: Gene Expression Omnibus
STRING: Search Tool for the Retrieval of Interacting Genes
PPI: Protein-protein interaction
BP: Biological processes
CC: Cellular component
MF: Molecular function
ROS: Reactive oxygen species
