# Supplementary material for: Expression profiling of spinal cord dorsal horn in a rat model of complex regional pain syndrome type-I uncovers potential mechanisms mediating pain and neuroinflammation responses
Source: J Neuroinflammation. 2020 May 23;17:162. doi: 10.1186/s12974-020-01834-0 (PMC7245895; doi:10.1186/s12974-020-01834-0)
Supplement: Supplementary file 10 — Additional file 10: Suppl. Table 10. Overlapping with SNI. [file 12974_2020_1834_MOESM10_ESM.docx]

**Suppl. Table 10. The 46 DEGs of CPIP overlapping with the SNI dataset**

| Genes symbol | Official Gene Name(NCBI) | Change in CPIP&SNI |
| --- | --- | --- |
| Cxcl13 | C-X-C motif chemokine ligand 13 | up |
| C3 | Complement component 3 | up |
| Reg3b | Regenerating family member 3 beta | up |
| C1qc | Complement C1q C chain | up |
| Cd68 | Cd68 molecule | up |
| C1qa | Complement C1q A chain | up |
| C1qb | Complement C1q B chain | up |
| Fcgr3a | Fc fragment of IgG receptor IIIa | up |
| Apobec1 | Apolipoprotein B mRNA editing enzyme catalytic subunit 1 | up |
| Irf8 | Interferon regulatory factor 8 | up |
| Fcgr2b | Fc fragment of IgG receptor IIb | up |
| Pld4 | Phospholipase D family, member 4 | up |
| Bin2 | Bridging integrator 2 | up |
| Ly86 | Lymphocyte antigen 86 | up |
| Ctsz | Cathepsin Z | up |
| Fcer1g | Fc fragment of IgE receptor Ig | up |
| Anxa3 | Annexin A3 | up |
| Tmem176a | Transmembrane protein 176A | up |
| Csf1r | Colony stimulating factor 1 receptor | up |
| Aif1 | Allograft inflammatory factor 1 | up |
| Tmem176b | Transmembrane protein 176B | up |
| Plek | Pleckstrin | up |
| Cd53 | Cd53 molecule | up |
| Laptm5 | lysosomal protein transmembrane 5 | up |
| Tgfb1 | transforming growth factor, beta 1 | up |
| Ifi30 | IFI30, lysosomal thiol reductase | up |
| Tyrobp | Tyro protein tyrosine kinase binding protein | up |
| Ctsh | cathepsin H | up |
| Clec4a3 | C-type lectin domain family 4, member A3 | up |
| Ptpn6 | Protein tyrosine phosphatase, non-receptor type 6 | up |
| Ctss | Cathepsin S | up |
| Tspo | Translocator protein | up |
| Cyba | Cytochrome b-245 alpha chain | up |
| Vav1 | Vav guanine nucleotide exchange factor 1 | up |
| Rac2 | Rac family small GTPase 2 | up |
| Klhl6 | Kelch-like family member 6 | up |
| Rnaset2 | Ribonuclease T2 | up |
| Grn | Granulin precursor | up |
| Slc7a7 | Solute carrier family 7 member 7 | up |
| Pycard | PYD and CARD domain containing | up |
| Arhgdib | Rho GDP dissociation inhibitor beta | up |
| Clic1 | Chloride intracellular channel 1 | up |
| Mpeg1 | Macrophage expressed 1 | up |
| Lcp1 | Lymphocyte cytosolic protein 1 | up |
| Ptprc | Protein tyrosine phosphatase, receptor type, C | up |
| Cd37 | CD37 molecule | up |
