# Supplementary material for: Expression profiling of spinal cord dorsal horn in a rat model of complex regional pain syndrome type-I uncovers potential mechanisms mediating pain and neuroinflammation responses
Source: J Neuroinflammation. 2020 May 23;17:162. doi: 10.1186/s12974-020-01834-0 (PMC7245895; doi:10.1186/s12974-020-01834-0)
Supplement: Supplementary file 11 — Additional file 11: Suppl. Table 11. Overlapping with CCI. [file 12974_2020_1834_MOESM11_ESM.docx]

**Suppl. Table 11.** **The 40 DEGs of CPIP overlapping with the CCI dataset**

| Genes symbol | Official Gene Name(NCBI) | Change in CPIP&CCI |
| --- | --- | --- |
| Cxcl13 | C-X-C motif chemokine ligand 13 | up |
| C3 | Complement component 3 | up |
| Reg3b | Regenerating family member 3 beta | up |
| C1qc | Complement C1q C chain | up |
| Cd68 | Cd68 molecule | up |
| C1qa | Complement C1q A chain | up |
| C1qb | Complement C1q B chain | up |
| Fcgr3a | Fc fragment of IgG receptor IIIa | up |
| Apobec1 | Apolipoprotein B mRNA editing enzyme catalytic subunit 1 | up |
| Irf8 | Interferon regulatory factor 8 | up |
| Fcgr2b | Fc fragment of IgG receptor IIb | up |
| Pld4 | Phospholipase D family, member 4 | up |
| Bin2 | Bridging integrator 2 | up |
| Ly86 | Lymphocyte antigen 86 | up |
| Ctsz | Cathepsin Z | up |
| Fcer1g | Fc fragment of IgE receptor Ig | up |
| Anxa3 | Annexin A3 | up |
| Tmem176a | Transmembrane protein 176A | up |
| Csf1r | Colony stimulating factor 1 receptor | up |
| Aif1 | Allograft inflammatory factor 1 | up |
| Tmem176b | Transmembrane protein 176B | up |
| Plek | Pleckstrin | up |
| Cd53 | Cd53 molecule | up |
| Laptm5 | lysosomal protein transmembrane 5 | up |
| Plac8 | Placenta associated 8 | up |
| C4a | Complement C4A | up |
| Cd33 | Cd33 molecule | up |
| Gpr183 | G protein-coupled receptor 183 | up |
| Fyb | FYN binding protein 1 | up |
| Atf3 | Activating transcription factor 3 | up |
| Clec7a | C-type lectin domain containing 7A | up |
| Gpr31 | G protein-coupled receptor 31 | up |
| Aoah | Acyloxyacyl hydrolase | up |
| Gapt | Grb2-binding adaptor protein, transmembrane | up |
| Cyth4 | Cytohesin 4 | up |
| Ltc4s | Leukotriene C4 synthase | up |
| Adgre1 | Adhesion G protein-coupled receptor E1 | up |
| Mx1 | Myxovirus (influenza virus) resistance 1 | up |
| Clec4a1 | C-type lectin domain family 4, member A1 | up |
| Fcgr1a | Fc fragment of IgG receptor Ia | up |
