# Supplementary material for: Expression profiling of spinal cord dorsal horn in a rat model of complex regional pain syndrome type-I uncovers potential mechanisms mediating pain and neuroinflammation responses
Source: J Neuroinflammation. 2020 May 23;17:162. doi: 10.1186/s12974-020-01834-0 (PMC7245895; doi:10.1186/s12974-020-01834-0)
Supplement: Supplementary file 12 — Additional file 12: Suppl. Fig. 1. Analysis of astrocyte activation, microglia activation and oligodendrocyte differentiation using GSEA. (A-C) GSEA analysis of CPIP model RNA-Seq dataset using well-defined gene sets for astrocyte activation, microglia activation and oligodendrocyte differentiation from the Molecular Signatures Database v7.1. (D-F) GSEA analysis of the SNI model dataset (GSE18803) for comparison with the CPIP model. [file 12974_2020_1834_MOESM12_ESM.pdf]

Suppl. Fig. 1

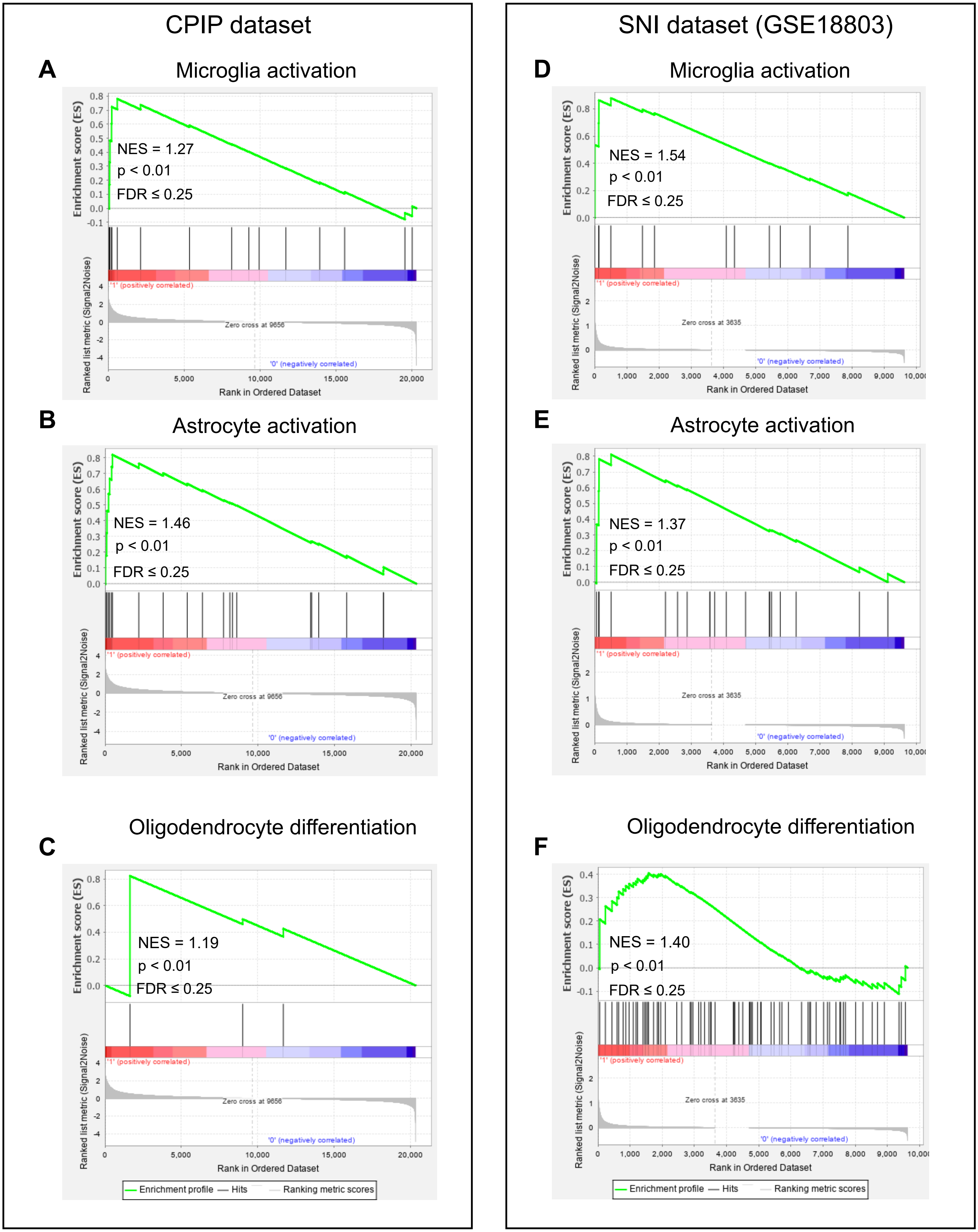
CPIP dataset
SNI dataset (GSE18803)
A
Microglia activation
NES = 1.27
p < 0.01
FDR ≤ 0.25
B
Astrocyte activation
NES = 1.46
p < 0.01
FDR ≤ 0.25
C
Oligodendrocyte differentiation
NES = 1.19
p < 0.01
FDR ≤ 0.25
D
Microglia activation
NES = 1.54
p < 0.01
FDR ≤ 0.25
E
Astrocyte activation
NES = 1.37
p < 0.01
FDR ≤ 0.25
F
Oligodendrocyte differentiation
NES = 1.40
p < 0.01
FDR ≤ 0.25
Enrichment score (ES)
Ranked list metric (Signal2Noise)
Rank in Ordered Dataset
'1' (positively correlated)
'0' (negatively correlated)
Zero cross at 9656
Zero cross at 3635
Enrichment profile
Hits
Ranking metric scores
